# Epidemic Investigation of the Jaundice Outbreak in Girdharnagar, Ahmedabad, Gujarat, India, 2008

**DOI:** 10.4103/0970-0218.66864

**Published:** 2010-04

**Authors:** Naresh T Chauhan, Prakash Prajapati, Atul V Trivedi, A Bhagyalaxmi

**Affiliations:** Department of Community Medicine, BJ Medical College, Ahmedabad, Gujarat, India

**Keywords:** Ahmedabad, epidemic investigation, jaundice, hepatitis E, outbreaks

## Abstract

**Background:**

Since 1976, seven outbreaks of hepatitis E occurred in Ahmedabad. Clusters of jaundice cases were reported on June 19, 2008, by a civic center, Girdharnagar ward, Ahmedabad.

**Objectives:**

The objectives were as follows: (1) to identify the etiological agent, source of outbreak, and mode of transmission; (2) to propose a control measure based on the outbreak investigation.

**Materials and Methods:**

We defined a case as an acute illness with (a) a discrete onset of symptoms and (b) jaundice or elevated serum aminotransferase levels, from March to September 2008 in the households of the Girdharnagar ward. We collected data through a door-to-door survey and hospital records. We described the outbreak in terms of time, place, and person. We collected laboratory investigation reports of case patients admitted to the civil hospital. To test our hypothesis we conducted a retrospective cohort study to find out the relative risk for hepatitis. We conducted environment investigation to find out the source of contamination of water supply.

**Results:**

A total 233 case patients of hepatitis were identified with the attack rate of 10.9/1000 population. Cases were reported in all the age groups with a higher attack rate in the age group of 20-29 years (18.5/1000). Out of 17 case patients, 16 were positive for the hepatitis E IgM antibody. The attack rate was two times more among those who were exposed to the leaking pipeline than the non-exposed (RR=2.3, 95% CI 1.76, 2.98). Environmental investigation also confirmed the sewage contamination of drinking water in the distribution system.

**Conclusion:**

The outbreak was due to hepatitis E virus. We recommended a temporary alternative water supply, repair of the leakages, and water quality surveillance.

## Introduction

Viral hepatitis caused by A and E viruses is the major public health problem in India.(1) Out of six different types of viral hepatitis known (A, B, C, D, E, and G), hepatitis E virus (HEV) is the agent responsible for the hepatitis outbreak as well as sporadic cases of hepatitis in developing countries.(1–3) Although hepatitis A and hepatitis E both are highly endemic in India, HEV infection is responsible for most of the outbreaks. In India, HEV infection is responsible for 30–70% of the cases of acute and sporadic hepatitis.(4) Since 1976 there were seven outbreaks of hepatitis E reported from Ahmedabad city.(2,5,6)

The virus is transmitted by the fecooral route, often through water or food supply contaminated by feces.(1,2,7) Intrafamilial transmission is not common for hepatitis E virus.(4,8) Acute viral hepatitis due to hepatitis E virus is a self-limiting disease.(7,9) The incubation period ranges from 2 weeks to 2 months, usually 1 month to 45 days.(2,10)

The recognition of early warning signals, timely investigation, and application of specific control measures can contain the outbreak and prevent death.(9) Recommendations based on the outbreak investigation also prevent future outbreaks.(2,10,11)

Clusters of jaundice cases were reported by the civic center run by the municipal corporation on June 19, 2008, in the Girdharnagar ward. All the initial cases were reported from the slum area near the civic centre, few of which were also admitted in Civil Hospital, Ahmedabad. A team consisting of residents of Community Medicine Department, BJ Medical College, Ahmedabad, investigated the outbreak of jaundice in the Girdharnagar ward of Ahmedabad city on June 21, 2008, to identify the causative agent the source of infection and to propose recommendations for future outbreaks.

## Materials and Methods

### Descriptive epidemiology

An epidemic investigation was carried out in the Girdharnagar ward having a population of 66,540 (census 2001). There were 21 *chawls* located around the Girdharnagar civic center, having a population of 21,363 affected by this outbreak. We reviewed the annual IDSP report on acute viral hepatitis to confirm the outbreak.(12) We searched cases by defining a case as an acute illness with (a) a discrete onset of symptoms and (b) jaundice or elevated serum aminotransferase levels, from March to September in the households of the Girdharnagar ward.(13) Data were collected through (1) a door-to-door survey and (2) hospital records. Information regarding the date of onset, age, sex, place of residence, treatment, and laboratory investigation was collected. The distribution of cases was analyzed using time, place, and person characteristics. Analysis was done using Epi Info 3.4.3 version.

### Analytical epidemiology

We conducted a retrospective cohort study to test the hypothesis regarding the cause of the hepatitis outbreak.(14) We divided the area into two cohorts on the basis of suspected exposer: (a) the area was supplied drinking water through leaking pipelines and there were overflowing drains; (b) the area was supplied with drinking water through pipelines without leakages. Then we identified people who developed the disease and who did not, among the exposed and non-exposed

### Laboratory methods

Blood samples of all 17 patients admitted to the Civil Hospital were subjected to serological tests for hepatitis A, B, C, and E.

### Environmental investigation

An investigation team visited houses and collected information regarding cases, water quality, source of water supply, and drainage system. Information regarding any public gathering exposure to outside food and local food vendor in March and April was also collected through a questionnaire. The available blueprint of the water supply pipelines and drains was examined.

## Results and Observations

**Figure 1:**
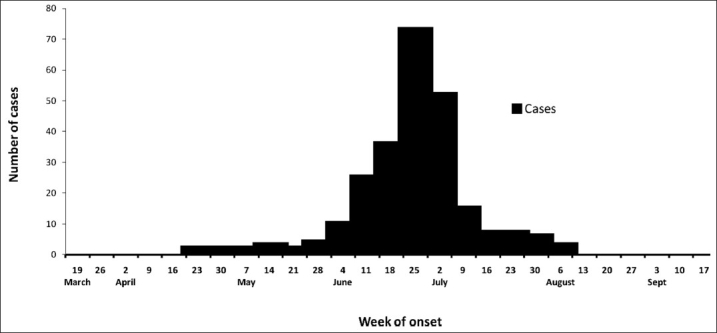
Cases of acute hepatitis by the week of onset, Girdharnagar ward, Ahmedabad, India, March–September 2008

**Figure 2:**
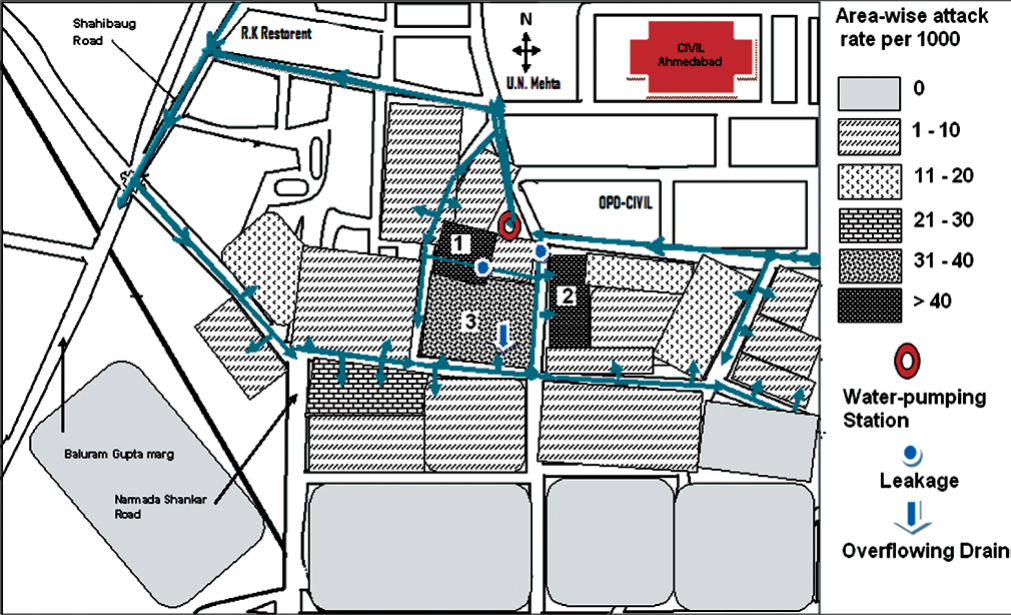
Incidence map of acute hepatitis by area in Girdharnagar, Ahmedabad, India, March–September 2008

The annual incidence of viral hepatitis as per the IDSP data ranged from 0.5 to 1.2 per 1000 in the Ahmedabad urban population during 2005–2006. The index case was reported to the Girdharnagar civic center on June 19 from the nearby slum. A total of 233 cases (attack rate 10.9/1000) were reported from March 2008 to September 2008. There was an initial cluster on the first week of June followed by a peak in the fourth week of June. The last case was reported on August 2, 2008 [Figure 1]. There were 151 males and 82 females affected of whom 1 female was pregnant. The disease affected all the age groups but the attack rate was highest among the age group of 20–29 years (18./ 1000) [Table 1]. The attack rate in males was 13/1000 and 8.3/1000 for females and the difference was statistically significant (*Z*=3.6, *P*<0.01). There was no death due to the disease. The area-wise attack rate was higher in areas 1 (44/1000), 2 (40/1000), and 3 (36/1000), Sarathi Apartment, Chimanlal Ghanchi, and Shantipura, respectively [Figure 2].

**Table 1:** Age- and sex-specific attack rate of jaundice cases in the Girdharnagar ward, Ahmedabad, India, 2008

	Group	Cases	Population	Attack rate per 1000 population	*Z*-value
Age (years)	0 to 9	22	4038	5.4	
	10 to 19	66	4444	14.9	
	20 to 29	75	4059	18.5	
	30 to 39	43	3204	13.4	
	40 to 49	17	2307	7.4	
	50+	10	3311	3.0	
Total		233	21,363	10.9	
Sex	Male	151	11,541	13.1	3.61
	Female	82	9822	8.3	(*P*<0.01)

The signs and symptoms included pain in abdomen (35%), anorexia (56%), malaise (32%), fever (39%), nausea (62%), vomiting (70%), icterus (100%), and yellow discoloration of urine (100%).

A higher incidence rate (16.3/1000) was observed among those who were exposed to leaking pipelines and defective drains compared to those non-exposed (7.1/1000). The relative risk for those exposed against those non-exposed was 2.3 (95% CI of RR 1.76, 2.98). The difference in the attack rate was also found to be statistically significant (c^2^ =41.1, *P*<0.001) [Table 2].

**Table 2:** Incidence rate in areas consuming water from leaking pipelines and having defective drains compared to those without the leakages and overflowing drains (Ahmedabad, India, March–September 2008)

Sources of water	Number of people affected	Number of people not affected	RR	95% CI	*P* value
Leaking pipes and overflowing drains (*n*=8838)	144	8694	2.3	1.76-2.98	<0.001
Area without leakages and Overflowing Drain (*n*=12,525)	89	12,436			

Total (*n*=21,363)	233	21,130			

A total of 16 out of 17 patients investigated were positive for the hepatitis E IgM antibody and one child was positive for the hepatitis A IgM antibody. Investigation reports of a private laboratory for 51 cases showed significantly higher SGOT, SGPT, and serum bilirubin.

The main source of water supply in Girdharnagar area is tap water supplied by the municipal corporation for 1–2 h in the morning. Complains of foul-smelling water initially and after the water supply were received from affected areas. Other possible sources had been ruled out. There was also a history of leakages in drinking water pipelines and overflowing drains in the area. Figure 2 shows the leakages and overflowing drains in the area. This finding was confirmed by the water supply management staff at the civic center. Residual chlorine was found in most of the water sample tested in various affected areas during the time of outbreak investigation. The timing of getting contaminated water supply coincided with the probable time period during which the possible exposure took place.

## Discussion

We identified 233 cases (attack rate 10.9/1000) during March–September 2008 in the Girdharnagar ward of Ahmedabad city, which was 10 times higher than that in previous data. We considered this unusual increase in jaundice cases as the outbreak of acute viral hepatitis. The epidemic of hepatitis E usually occurs in the unimodal outbreak with a highly compressed curve of incidence or is a prolonged epidemic with multiple peaks.(5) In our study, it was a unimodal outbreak with a single peak suggestive of a point-source, common-vehicle epidemic. The age specific incidence was highest among 20–29 years age group (18.5/1000), similar to what was reported by another study.(15–18)

The results of this investigation indicated that the outbreak was caused by the hepatitis E virus. The factors which contributed to this outbreak were leakages in drinking water pipelines and overflowing drains. The outbreak was subsided after correction of these factors. There were seven outbreaks of hepatitis E documented from Ahmedabad city since 1976 to 2005 and 115–2572 cases were reported in these outbreaks.(5,6) However, they involved different areas of the city at that time.

This outbreak occurred in the slum area receiving tap water supply from the municipal corporation only for 2 h in the morning. The present outbreak was waterborne as indicated by this study. Findings of the cohort study indicated that those who drank water from leaking water pipeline were at an increased risk of hepatitis (RR=2.3, 95% CI 1.76, 2.98). A similar observation was made by Banerjee *et al*. in their study.(14) The termination of the outbreak following the extensive repair work carried out by the corporation further supported our hypothesis.

Hepatitis E outbreaks have been reported in urban areas whenever there is a break in the quality of water supplied including water chlorination.(9,14,15) Several studies had similar epidemiological features. Poor sanitation and densely populated slum favor not only the hepatitis E outbreak but also typhoid and cholera outbreak.(3,19) Fecal contamination of the source of drinking water was documented in many of the epidemics.(3,14,15,17–19)

Water samples were not tested for the coliform count which was the limitation of our study and which could have further supported our analytic findings in this study.

The outbreak that affected the Girdharnagar ward was caused by waterborne viral hepatitis E. The most likely source of the outbreak was drinking water contaminated with sewage due to leakages aggravated by overflowing drains and intermittent water supply. Based on our findings in this study, we recommend an alternative arrangement for water supply for the time being whenever there are complains of getting dirty water; repair of the leakages in the water pipelines as early as possible; and regular testing of residual chlorine and coliform count from different distribution points of water supply.
